# Design of Chitin Cell Culture Matrices for 3D Tissue Engineering: The Importance of Chitin Types, Solvents, Cross-Linkers, and Fabrication Techniques

**DOI:** 10.3390/pharmaceutics16060777

**Published:** 2024-06-07

**Authors:** Turna Basak, Julia L. Shamshina

**Affiliations:** Fiber and Biopolymer Research Institute, Department of Plant and Soil Science, Texas Tech University, Lubbock, TX 79403, USA; tbasak@ttu.edu

**Keywords:** biopolymers, chitin, tissue engineering, scaffold, chitin TE scaffolds preparation

## Abstract

This review focuses on factors and the fabrication techniques affecting the microarchitecture of tissue engineering scaffolds from the second most abundant biopolymer, chitin. It emphasizes the unique potentiality of this polymer in tissue engineering (TE) applications and highlights the variables important to achieve tailored scaffold properties. First, we describe aspects of scaffolds’ design, and the complex interplay between chitin types, solvent systems, additives, and fabrication techniques to incorporate porosity, with regard to best practices. In the following section, we provide examples of scaffolds’ use, with a focus on in vitro cell studies. Finally, an analysis of their biodegradability is presented. Our review emphasizes the potentiality of chitin and the pressing need for further research to overcome existing challenges and fully harness its capabilities in tissue engineering.

## 1. Introduction

There is an increased demand to develop scaffolds that mimic the extracellular matrix (ECM) for tissue engineering (TE) applications, to culture viable human tissues outside the body. The ECM is a three-dimensional (3D) network that serves as a physical scaffold for cells [1] and is a part of all our body’s tissues. It consists of mostly two main types of macromolecules: proteoglycans (PGs) that have core proteins in their structure, with side glycosaminoglycan (GAG) chains, and fibrous proteins such as collagen, elastin, and fibronectin. These entangled macromolecules form the structurally stable 3D network and are particularly abundant in cartilaginous tissues [2].

In addition to contributing to the mechanical properties of tissues, the ECM is an anchorage site for cells and a repository of growth factors (insulin-like growth factor (IGF), platelet-derived growth factor (PDGF), fibroblast growth factors (FGF), transforming growth factor-β (TGF-β), hepatocyte growth factors (HGF), etc.) that are required for the protection of cells from degradation and sustaining their appropriate size as they proliferate. ECM is also a reservoir of bioactive molecules. For instance, in the central nervous system (CNS), ECM offers structural support to neurons and glial cells and helps maintain balance in ionic and nutritional conditions [3]. ECM actively participates in the remodeling and development of the kidney via cellular communication pathways and aids in the renewal of damaged cells with new ones during tissue repair [4].

The ECM’s composition and structure are different for different tissues. When developing 3D tissue models, simulating the ECM’s properties in artificial materials is important for delivering an appropriate environment for cell growth. From a material engineering perspective, artificial materials must provide relatively strong structural support for cells [5]; cells must not only adhere to the artificial network but also be able to grow, migrate, and proliferate until they can perform in a way similar to the surrounding tissues [6]. The artificial scaffolds should be mechanically strong so that their mechanical properties (toughness, rigidity, and elasticity) match those required at the implanted site.

Artificial scaffolds must be biocompatible and must produce a minimal immune reaction. They also must be biodegradable as they represent only temporary cell support, which is no longer needed when ECM replaces the implanted structures. It is essential to achieve the required porosity and permeability of the 3D network, to allow for nutrient and metabolite transport. Several types of both natural and synthetic matrix gels that simulate ECM, promote cell adhesion and proliferation, and possess suitable transport properties are known (e.g., collagen, Matrigel^®^, fibrin gel [7], hyaluronic acid gel [8], polyethylene glycol- (PEG-)based hydrogels [9]), etc.

**Figure 1:**
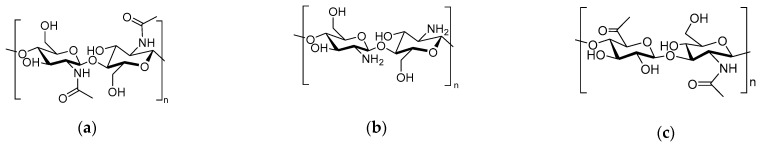
Structures of chitin (**a**), chitosan (**b**), and glucosamine glucan (**c**).

The design of scaffolds is the core of TE, and today the choice of materials for scaffolds has shifted toward natural systems. Among these, chitin and its derivative, chitosan, are suitable substrates that modulate cell behavior during tissue regeneration, through unique interactions with GAG and PG components in the ECM [6]. Chitin and chitosan are linear polysaccharides made up of different proportions of 2-amino-2-deoxy-D-glucopyranose (GlcN) and 2-acetamido-2-deoxy-D-glucopyranose (GlcNAc) units, connected through *β*-(1→4) linkages [10]. The percentage of –NAc groups is known as the degree of acetylation (%DA). Chitin (Figure 1a) is made of >50% GlcNAc units (i.e., %DA > 50%), while chitosan (Figure 1b) is made of >50% GlcN units (i.e., %DA < 50%); the similarities of these two polymers to the structure of GAGs are shown in Figure 1c.

Chitosan applications in the TE area are well documented and numerous reviews cover the use of chitosan in regenerative medicine [11,12]. However, the biological properties of chitin and chitosan differ. Thus, both chitin and chitosan exhibit excellent biocompatibility, but this property is greatly affected by the polymers’ molecular weight (Mw) and %DA. Each polymer possesses a spectrum of other beneficial properties, including analgesic, antitumor, hemostatic, hypocholesterolemic, and antioxidant [13,14,15], as well as hemostatic efficacy [16], which is the first stage of wound healing [17].

Both polymers are efficient immunomodulators [11]. Chitin stimulates pathogens’ uptake by macrophages and dendritic cells via lectin binding [11]. Post-translational modification of proteins with chitin is also a cell-signaling mechanism that controls various aspects of cell function. Chitosan stimulates macrophages to produce cytokines and other compounds to improve non-specific host resistance against bacterial and viral infections; it also stimulates depression of adaptive type-2 allergic immune response. The antibacterial activity of chitin and chitosan depends on the bacterial strain, the nature of the antibacterial mechanism, and Mw. The minimum inhibitory concentration (MIC) value ranges from 0.006% to 0.100% for chitin, and 0.006% to 0.030% for chitosan [18]. Both polymers promote wound healing [19]. It was determined that a lower %DA stimulated fibroblast proliferation but inhibited human keratinocyte growth [20]. Chitin is suitable for cellular attachment, proliferation, and differentiation [21] and promotes the proliferation of fibroblasts, dermal granulation, and vascularization. Chitin also plays a vital role in bone mineralization, for bone repair (i.e., in chitin–calcium phosphate composites).

In addition, the biodegradation rate depends on %DA, repeat unit order, and Mw [22]. The degradation increases with an increase in %DA, and for chitin, reported degradation in the human body is known to be 12 weeks post-surgery (exemplified with implanted chitin fabric). Chitin degradation is enzymatic, under the actions of both lysozymes and chitinases; chitosan undergoes degradation under the action of chitinases [22].

Concerning physical properties, chitosan is more hydrophilic than chitin and to prevent it from absorbing water, it should be laminated, or composed with other polymers [23]. Chitosan’s low mechanical integrity also necessitates its composition or co-grafting with natural, semisynthetic, or synthetic polymers in TE applications [24]. For instance, in cartilage tissue engineering, silk fibroin has been employed alongside chitosan [25]. In bone tissue engineering, chitosan has been paired with Nylon-6,6 [26]. For respiratory tissue engineering, the combination of chitosan and poly(Ɛ-caprolactone) was used to impart structural integrity [27]. In addition, chitosan impedes the migration and mobility of specific cell types, which is a crucial aspect of tissue development [28].

On the other hand, chitin has remarkable mechanical properties and can be used alone, with no copolymers and/or grafting, to construct tissue engineering matrices. Numerous cell culture experiments have shown the capability of these matrices to support robust cell growth and proliferation, suggesting their potential application in cell transplantation for tissue regeneration [29].

Engineering techniques for TE scaffolds are focused on precise control of the microarchitectural features within the construct. Porousness and pore size of the scaffold are important factors for cell seeding and growth. With chitin polymer, it is possible to control the intricate structural features of the scaffolds, preserve interconnected pores, control the distribution and morphology of the pores, and optimize them for various regenerative purposes. These features made chitin scaffolds one of the major scaffold types with a broad spectrum of applications [28]. To illustrate, chitin is used in soft TE engineering applications (in combination with e.g., poly(butylene succinate)/chondroitin sulfate nanoparticles [30], hyaluronic acid [31], bone TE applications (in combination with e.g., nanosilica [31], hydroxyapatite [32], or poly(ε-caprolactone) [33], or vascular TE applications [34].

This review focuses on the preparation of TE scaffolding materials from chitin. While research on chitin-based scaffolds has been extensive, it has lacked a systematic approach. Unlike synthetic polymers, chitin is obtained from various natural sources, resulting in variations in the polymer characteristics, including molecular weight (Mw) and degree of acetylation (%DA), causing inconsistencies in the polymer’s biological properties. In addition, different protocols have been employed to prepare chitin scaffolds. These protocols use diverse forms of chitin (micro-, macro-, or nanocrystalline), various chitin amounts, and different solvents. While some materials are prepared without cross-linking, others use cross-linkers of different types. The methodologies also employ various fabrication techniques to introduce porosity into materials. This contributes to the intricate and multifaceted nature of chitin-based scaffolds and affects material porousness, pore size, surface area, and other microarchitectural features of chitin constructs and, respectively, the performance of materials. In this review, we address these questions individually.

## 2. Chitin Types

Chitin ((C_8_H_13_O_5_N)_n_) ranks as the second most prevalent biopolymer [34], following cellulose. This natural polysaccharide is prominent in the exoskeletons of different arthropods such as insects, crustaceans, and arachnids [35]. It is also found in the cell walls of fungi and other microorganisms.

**Figure 2:**
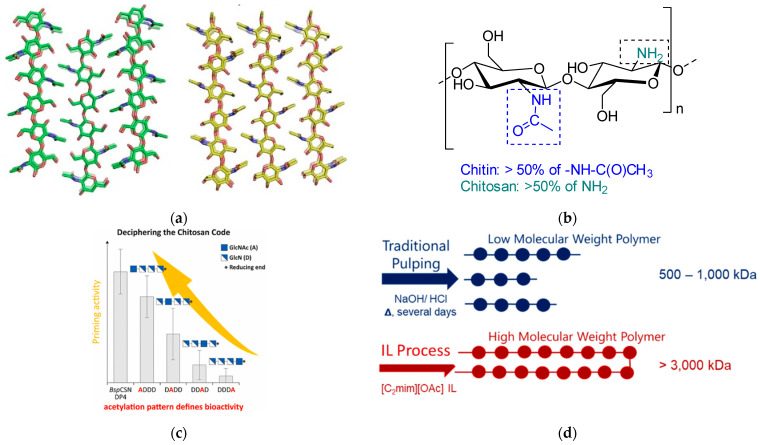
(**a**) Molecular models of the crystal structure of *α*-chitin (**left**) and *β*-chitin (**right**) showing the antiparallel and parallel chain directionality. Reprinted with permission from ref [38]. Copyright © 2021 The Royal Society Publishing; (**b**) Structure of chitin vs. chitosan; (**c**) Pattern of acetylation (PA): the sequence of *β*-1,4-linked glucosamine (deacetylated) and *N*-acetylglucosamine (acetylated) units. Reprinted with permission from ref. [41]. Copyright © 2020 American Chemical Society; (**d**) Schematic representation of high- and low-molecular-weight (Mw) chitin [42,43,44].

Depending on the crystalline structure, three distinct allomorphic forms of chitin are found in nature: *α*-chitin, *β*-chitin, and *γ*-chitin [36] (Figure 2a). The *α*-chitin allomorph contains polymeric chains arranged in an anti-parallel fashion, and has extensive inter- and intramolecular hydrogen bonding; it is the predominant polymorphic form present in crustaceans, as well as in the cell walls of fungi [35,37]. Because of extensive inter- and intramolecular hydrogen bonding, *α*-chitin is difficult to dissolve. The *β*-chitin allomorph encompasses polymeric chains arranged in a parallel fashion and contains a smaller number of intermolecular hydrogen bonds, resulting in higher solubility than that of *α*-chitin [38]. The *β*-chitin allomorph is found in squid pens, within the cell walls of diatoms, and in skeletal structures of cephalopods. The third allomorph of chitin, *γ*-chitin, is a mixture of *α-* and *β*-chitin, where every third polymeric chain follows the opposite direction to the previous chains [39]. TE scaffolds have been prepared from both *α*-chitin (Table 1, **Entries 3**, **4**, **6**–**8**, **12**–**20**, and **22**–**24**) and *β*-chitin (Table 1, **Entries 1**, **2**, **5**, **9**–**11**, **21**) underscoring the variability inherent to this chitin type within scaffolding materials.

The polymer can be used either in a microcrystalline form (“bulk” chitin) or nanocrystalline form (nanochitin). The physicochemical and biological properties of chitin vary from the nanoscale to the microscale [40]. In turn, nanochitin is categorized into chitin nanofibers (ChNFs), chitin nanocrystals (ChNCs), or chitin nanowhiskers (ChNWs). Both bulk chitin (Table 1, **Entries 1**–**24**) and nanochitin (Table 1, **Entries 25**–**27**) have been utilized to prepare chitin scaffolds.

It is also crucial to acknowledge the significance of the degree of acetylation (%DA) and the DA pattern in chitin (Figure 2b,c, respectively). As mentioned previously, the %DA, representing the percentage of acetyl groups anchored to the glucosamine units, influences key physicochemical properties such as hydrophobicity, crystallinity, and degradation rate [41]. As this review covers TE scaffolds made from chitin, only examples with %DA > 50% have been included in Table 1.

Key considerations in the selection of the type of chitin for biomedical scaffold fabrication encompass a spectrum of factors, including but not limited to the form and molecular weight of the chitin polymer. The Mw of chitin (Figure 2d) differs based on the biomass it was isolated from, methods that were used for chitin isolation, and the species’ age/size, as was shown for shrimp [43]. Specifically, the traditional method of chitin isolation (“pulping”) that requires demineralization and deproteinization using acids and bases, respectively, results in relatively low Mw polymer. This is a commercially available polymer, also called practical grade (PG) chitin. High-molecular-weight chitin is obtained through extraction using 1-ethyl-3-methylimidazolim acetate ([C_2_mim][OAc]) ionic liquid (IL) [42,44]. IL-extracted chitin has been shown to have Mw at least ~2.5 times exceeding that of “pulped” chitin on average (~3.9 MDa vs. ~1.6 MDa). When small-size shrimp (5–10 and 10–20 g average body weight) were used as a chitin source, the Mw of extracted chitin was found to be ∼4–5 MDa, whereas more mature larger size shrimp (>30 g average body weight) provided the polymer with a significantly higher Mw of 23 MDa [43]. There are also “pulping” methods available for chitin isolation using ILs (e.g., hydroxyethylammonium acetate [HOCH_2_CH_2_NH_3_][OAc] IL) [45,46] that provide chitin with Mw higher than that of the polymer obtained with the traditional pulping process but lower than that of the polymer extracted with the help of [C_2_mim][OAc].

## 3. Types of Solvents

**Figure 3:**
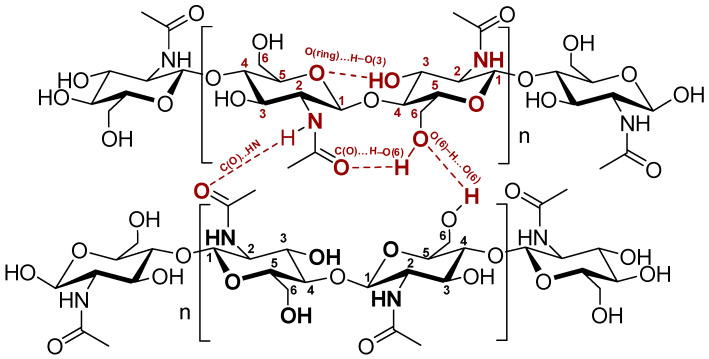
Hydrogen bonding network in α-chitin.

Chitin dissolution is the only feasible option for processing the biopolymer in its natural form without derivatization. The challenge of processing the polymer is its insolubility in water and conventional organic solvents. One of the main reasons is the highly crystalline structure of chitin, fostering intra- and intermolecular hydrogen bonds within its macromolecules (Figure 3).

When “typical” organic solvents are employed, the β-chitin polymer swells [36,47,48], rather than being dissolved in a solvent. β-chitin conformation is more prone to intra-crystalline swelling than α-chitin conformation. Swelling with water, alcohol, and amines via intra-crystalline swelling in the β-conformation takes place without disrupting the sheet organization and the crystallinity, whereas water and alcohols cannot penetrate the crystalline lattice of α-chitin.

For chitin dissolution to happen, there is a need to break the native hydrogen-bonded network. This requires disassembling its supramolecular structure (total four hydrogen bonds per one *N*-acetylglucosyl unit, with two intra- and two intermolecular hydrogen bonds in *α*-chitin, Figure 3) and separation of the polymer chains without or with the minimum breakage of glycosidic bonds.

Although some studies revealed certain solvents (discussed below) can dissolve chitin, many of these solvents present drawbacks associated with health (toxicity, mutagenicity), chemical handling (corrosiveness, waste disposal), and poor degradability. Extensive reviews have covered the topic of chitin dissolution mentioning traditional solvent systems capable of disrupting the polymer hydrogen bonding [47,49], which include inorganic bases (e.g., sodium hydroxide (NaOH)), calcium salts (e.g., calcium thiocyanate (Ca(CNS)_2_), calcium iodide (CaI_2_), calcium bromide (CaBr_2_), or calcium chloride (CaCl_2_)), and lithium salts (e.g., lithium thiocyanate (LiCNS)) [50]. These solvents are not extensively used for TE applications due to the difficulty of their removal [50].

Halogenated (perfluorinated or chlorinated) solvent systems are also used for chitin dissolution. These systems include 2-chloroethanol, 1-chloro-2-propanol, 2-chloro-1-propanol, and 3-chloro-1,2-propanediol in combination with sulfuric acid [51], although the use of these solvents results in chitin degradation to a significant extent, due to acid-catalyzed depolymerization. Perfluorinated 2,2,2-trifluoroethanol and trichloroacetic acid mixtures with chlorinated hydrocarbons represent other solvent systems for chitin [52,53]. A system of formic acid–dichloroacetic acid–isopropyl ether has also been employed for chitin dissolution [54,55]. Such systems cannot be used for TE purposes due to high perfluorination or chlorination and, therefore, associated toxicity.

Another solvent system, CaCl_2_–methanolic solution is extensively used for TE applications [56]. This is, perhaps, the most often utilized system for manufacturing TE scaffolds from chitin (Table 1, **Entries 1**–**10**). It is suitable for making scaffolds not only from individual polymers but also from their composites with nanoscale molecular compounds (e.g., nanosilver [57], nanodiopside [58], nanohydroxyapatite [58], nanobioactive glass [59], nanosilica [31], CaCO_3_ nanopowder [60]), natural polymers (e.g., silk fibroin [61], pectin [60], chitosan [60], gelatin [62]), and polyhydroxyalkanoates (e.g., poly(3-hydroxybutyrate-co-3-hydroxyvalerate PHBV [63]).

The mechanism of chitin dissolution in CaCl_2_–methanolic solution involves an association of calcium cations (Ca^2+^) with –OH groups of chitins forming a chelate which disrupts H-bonding [64,65]. It has also been suggested that Ca^2+^ attacks the amide bond in the chitin side chains, disrupting the tight crystalline network [66]. During the dissolution process, the presence of calcium cations in a highly concentrated salt solution hampers the transparency of the chitin solution, diminishing its transmittance [66]. It has also been reported that the dissolution of chitin in this system depends on the amounts of water and Ca^2+^ cation. In addition, the solubility of chitin is also affected by %DA, as well as Mw [66]: the lower the %DA and Mw, the higher the polymer’s solubility.

Another typical system is *N*,*N*-dimethylacetamide (DMAc) used in combination with lithium chloride (LiCl), see Table 1, **Entries 11**–**19**. The amount of LiCl in the DMAc is typically 5–8% (*w*:*v*). The factors influencing the dissolution include concentration, time, and temperature. Here, the Li^+^ cation of LiCl salt coordinates with the carbonyl groups of DMAc, forming a complex in which the lithium cation is strongly bound to the amide carbonyl oxygen, and the chloride anion is thus involved in the disruption of the chitin hydrogen bonds. This was shown in the example of the dissolution of cellulose, a biopolymer structurally similar to chitin, via ^13^C NMR studies revealing the strong interaction of the LiCl molecule with intermolecularly hydrogen-bonded hydroxyls. In the case of chitin, ^1^H NMR investigations demonstrated the strong interaction of the LiCl molecule not only with intermolecularly hydrogen-bonded hydroxyls but also with acetamide groups [67].

Two of the vital disadvantages of the DMAc/LiCl solvent system are its hygroscopicity and cost. Both components of the system are highly hygroscopic and have to be protected from air moisture since the dissolution ability of the solvent is significantly reduced with the absorption of water, resulting in polymer aggregation; the same issue is known for cellulose [68]. The absorption of water into the solvation shell of the Li^+^ cation hydrolyzes DMAc and initiates the uptake of additional water molecules, but an even more important challenge is the formation of a highly reactive keteniminium cation from DMAc at temperatures exceeding 80–85 °C. The keteniminium cation promotes the glycosidic cleavage of any biopolymer [69], including chitin, decreasing its Mw.

Methods using sodium hydroxide–urea aqueous eutectic (8 wt% NaOH/4 wt% urea/88% water) have also been used extensively for the preparation of chitin materials since 2007 [70], at low temperatures, employing freeze–thawing cycles [71,72]. Examples of TE materials prepared using this solvent system are provided in Table 1, **Entries 20–22**. There, urea was suggested to play an important role in the chitin solution stability [73]. It must be noted that chitin aqueous solution is susceptible to temperature changes and forms a gel upon temperature increase. It has also been shown that chitin chains in solution accommodate a random coil conformation [73]. The proposed dissolution mechanism of chitin in NaOH–urea aqueous solution [74] suggests a three-step process: 1. The formation of the hydrogen bond network between NaOH and chitin chains; 2. The formation of the H-bonds between the hydroxide of NaOH and urea; and 3. Attachment of the urea–hydrate clusters to the surface of the NaOH hydrogen-bonded chitin. The resulting complex is water-soluble.

In addition to TE scaffolds from pure chitin [75], sodium hydroxide–urea aqueous eutectic was suitable for the preparation of composites with synthetic polymers such as poly(ε-caprolactone) (PCL) and biopolymers such as hyaluronic acid (HA) [76]. The method has also been used for the derivatization of chitin using 1,2-propylene epoxide (PO), to form hydroxypropyl chitin, before its modification into a scaffold [77]. The disadvantage of this solvent system is that it could lead to partial chitin deacetylation due to the presence of NaOH [78]. Furthermore, the solvent is unable to dissolve high-Mw polymer [44], whereas proper control over the microstructure requires the complete dissolution of chitin, without degradation.

Ionic liquids (ILs) made of an imidazolium cation paired with a strongly basic, hydrogen-bond-accepting anion (e.g., acetate ([OAc]^−^, formate [HCOO]^−^, chloride Cl^−^, methyl phosphate [(MeO)HPO_2_]^−^, dimethyl phosphate [(MeO)_2_PO_2_]^−^), have been applied in the preparation of chitin solutions. The most common ILs for chitin dissolution include 1-allyl-3-methylimidazolium chloride ([Amim]Cl]) and acetate ([Amim]OAc]), 1-butyl-3-methylimidazolium chloride ([C_4_mim]Cl) and acetate ([C_4_mim]OAc]), 1-ethyl-3-methylimidazolium chloride ([C_2_mim]Cl]) and acetate ([C_2_mim]OAc]), etc. [46]. There are only two examples in which this type of solvent was used for TE scaffold formation (Table 1, **Entries 23**–**24**).

Deep eutectic solvents (DESs) that do not contain entirely ionic species exhibit physicochemical properties similar to those of ILs and can dissolve chitin as well. Traditional DESs are prepared from choline chloride and urea or thiourea, forming DESs with an mp of 12 °C or 69 °C, respectively [79]. To summarize, the careful selection of an appropriate solvent for chitin solubilization stands as a primary consideration in both laboratory-scale research and industrial scaling practices [80] for TE.

**Table 1:** The morphological properties of chitin scaffolds, depending on the method of preparation.

#	Type of Solvent	Type of Chitin	Additional Components	Cross-Linkers	Drying Techniques	Type of Scaffold	Morphological Properties			Ref.
							Porosity, %	Pore Size	Area, m^2^/g	
1	Methanolic CaCl_2_	*β*-Chitin (%DA 72.4%)	Nanosilver	None	Lyophilization (no T of freezing provided)	*β*-Chitin–nanosilver	ND	~500 µm ^f^	ND	[81]
2		*β*-Chitin	Nanodiopside, nanohydroxyapatite	Glutaraldehyde	Lyophilization after freezing at −80 °C	*β*-Chitin–nanodiopside–nanohydroxyapatite	67–81 ^g^	126–400 µm ^h^	11.24	[58]
3		*α*-Chitin	Nanobioactive glass ceramic	None	Lyophilization after freezing at −80 °C	*α*-Chitin–nanobioactive glass ceramic	ND	150–500 µm	ND	[59]
4		*α*-Chitin	Nanosilica	None	Lyophilization (no T provided)	*α*-Chitin–nanosilica	ND	ND	ND	[31]
5		*β*-Chitin	Hydroxyapatite	None	Lyophilization after freezing at −20 °C	*β*-chitin–nanohydroxyapatite	70–80	ND	ND	[82]
6		*α*-Chitin (%DA 75.6%)	Hydroxyapatite	None	Lyophilization after freezing at −20 °C	*α*-Chitin hydrogel–nanohydroxyapatite	72–79 ^i^	250–400 µm	ND	[57]
7		*α*-Chitin (%DA > 72.4%)	Silk fibroin	Glutaraldehyde	Lyophilization after freezing at −20 °C	*β*-Chitin–silk fibroin	76–81	ND	ND	[61]
8		*α*-Chitin	Pectin, CaCO_3_ nanopowder	Chitosan	Lyophilization (no T of freezing provided)	*α*-Chitin–pectin–CaCO_3_ nanopowder	~42	200–300 µm	ND	[60]
9		*β*-Chitin	Gelatin, hydroxyapatite	Glutaraldehyde	Lyophilization after freezing at −80 °C	*β*-Chitin–gelatin–nanohydroxyapatite	68–81 ^j^	126–400 µm ^j^	ND	[62]
10		*β*-Chitin (%DA 85%)	PHBV ^a^	None	Lyophilization (no T of freezing provided)	*β*-Chitin–PHBV	67	<20 µm	ND	[63]
11	DMAc/ 5% LiCl	*β*-Chitin	Atelocollagen	UV irradiation	Lyophilization after freezing at −75 °C	*β*-Chitin–collagen	63–78 ^k^	241–429 µm ^k^	ND	[83]
12		*α*-Chitin	Hydroxyapatite	None	Lyophilization after freezing at −38 °C	α-Chitin–hydroxyapatite	69	200–400 µm	ND	[32]
13		*α*-Chitin	Sugar	None	Lyophilization (no T of freezing provided)	*α*-Chitin	ND	500 µm	ND	[84]
14		*α*-Chitin (%DA > 75%)	None	None	Supercritical CO_2_ (sc-CO_2_)	*α*-Chitin	83–92 ^l^	2–50 nm	205–365 ^l^	[85]
15		*α*-Chitin	None	None	Lyophilization after freezing at −20 °C	Chitin	53.9	10 µm	ND	[86]
16		*α*-Chitin	None	None	Lyophilization after freezing at −196 °C	*α*-Chitin	61.2	100–200 µm	ND	[86]
17		*α*-Chitin	None	None	Lyophilization after freezing at −38 °C	*α*-Chitin	68.8	200–500 µm	ND	[86]
18		*α*-Chitin	None	None	Supercritical CO_2_ (sc-CO_2_)	*α*-Chitin	9.8	ND	ND	[86]
19		*α*-Chitin	None	None	Air drying	*α*-Chitin	12.9	ND	ND	[86]
20	NaOH/ Urea	*α*-Chitin	None	None	Sc-CO_2_	*α*-Chitin	ND	ND	<366	[75]
21	NaOH Solution	*β*-chitin sponge	None	None	Lyophilization (no T of freezing provided)	Cartilage–scaffold composites	ND	100–200 µm	ND	[87]
22		*α*-Chitin	*β*-glucan	None	Materials were studied as hydrogels (not dried)	Fungal mycelial mats with chitin–glucan polysaccharide cell walls	53–63	ND	ND	[88]
23	Ionic Liquid	*α*-Chitin (%DA ~58%)	Sucrose acetate isobutyrate	None	Lyophilization after freezing at −77 °C	*α*-Chitin–sucrose acetate isobutyrate	44–89	57–106 µm	ND	[89]
24		*α*-Chitin	None	None	Supercritical CO_2_ (sc-CO_2_)	Chitin	84–90	2–50 nm	108–145	[90]
25	Aqueous suspension	Chitin nanocrystals	POFC ^b^	Thermo cross-linking	Lyophilization after freezing at −50 °C	Chitin–nanocrystals–POFC	~80	ND	ND	[28]
26		Chitin nanocrystals	PHBV, ^a,d^ NaCl (porogen)	None	Dried at 25 °C ^d^	Chitin nanocrystals–PHBV	ND	9.6 µm	ND	[91]
27		Chitin nanocrystals	Hyaluronan, gelatin	EDC ^c^	Lyophilization after freezing at −50 °C	Chitin–hyaluronan–gelatin	ND	92–230 µm ^e^	ND	[92]

^a^ PHBV = poly(3-hydroxybutyrate-co-3-hydroxyvalerate; ^b^ POFC = poly(1,8-octanediol-coPluronicF127)citrate; ^c^ EDC = 1-ethyl-3-[3-dimethylaminopropyl]carbodiimide hydrochloride (EDC); ^d^ The main component of the scaffold was PHBV that does not collapse upon air drying. The amount of chitin was 10% of PHBV; ^e^ Different sizes in the transverse and longitudinal cut. Sizes depended on chitin load (0–30%); ^f^ Obtained from SEM images; ^g^ Different weight ratios of *β*-chitin–nanodiopside–nanohydroxyapatite (30/15/55, 50/11/39, 70/6.5/23.5) produced materials with different porosity (%); ^h^ Different weight ratios of β-chitin–nanodiopside–nanohydroxyapatite (30/15/55, 50/11/39, 70/6.5/23.5) produced materials with different pore size; ^i^ Values extracted from the graph; ^j^ The porosity and pore sizes depended on the ratios of chitin–gelatin–hydroxyapatite (15/15/70, 25/25/50, 35/35/30); ^k^ Different weight ratios of NaCl–*β*-chitin (100–700 g/g) produced different sizes of pores and porosity; ^l^ The porosity and surface area depended on the drying pressure (from 80 to 300 bar) and temperature (from 40 to 80 °C).

## 4. Cross-Linkers

To achieve the desired mechanical properties of scaffolding materials, 3D biomaterials are engineered with the aid of exogenous compounds known as cross-linkers [93]. An exemplary cross-linked material should possess a range of crucial attributes, including enhanced mechanical properties, non-toxicity, active interaction with cells, reduced gas permeability, and the ability to maintain structural integrity [94]. The increased resistance to enzymatic degradation may be a disadvantage for TE scaffolds.

Types of cross-linking are typically categorized into two groups: physical cross-linking (e.g., van der Waals forces, hydrogen bonding, or ionic interactions) and chemical cross-linking (covalent bonds) [95,96]. Among physical crosslinking techniques, chitin and collagen can be cross-linked via dehydrothermal (DHT) treatment, γ-irradiation, and ultraviolet (UV) irradiation. DHT treatment offers the advantage of simultaneous cross-linking and sterilization, thereby reducing immunogenic responses and enhancing cellular activity, leading to improvements in scaffold pore size, swelling kinetics, and cellular metabolic function [96,97,98]. UV irradiation facilitates the formation of bonds between polypeptide chains without affecting their acidic and basic side chains, while also sterilizing the material via disrupting nucleic acid integrity, thereby enhancing cell recognition sites [99,100]. For instance, as indicated in Table 1, **Entry 11**, the physical cross-linking of *β*-chitin and *β*-chitin–collagen scaffold using UV irradiation resulted in improved mechanical strength and adequate porosity. Collagen materials exposed to UV irradiation are usually physically cross-linked by placing material into the UV cross-linking chamber and exposing them to a bank of 15 W UV bulbs for 15 to 240 min. Physical cross-linking is accomplished through inducing physical and chemical changes in type I collagen [101]. Utilization of electrostatic interaction using sodium citrate (SC), tripolyphosphate (TPP), or hydrophobic interaction using β-glycerophosphate is more typical for chitosan cross-linking, due to the presence of cationic [R-NH_3_]^+^ groups.

Chemical cross-linkers, such as glutaraldehyde, N-hydroxy-succinimide (NHS), 1,2,3,4-butanetetracarboxylic dianhydride (BTCA), succinic anhydride (SA), citric acid (CA) epichlorohydrin (ECH), ethylene glycol diglycidyl ether (EGDE), divinyl sulfone (DVS), and 1-ethyl-3-[3-dimethylaminopropyl]carbodiimide hydrochloride (EDC), are widely employed for their ability to enhance degradation resistance and stabilize chitin materials. Not all of them have been used for the preparation of scaffolds. Examples of cross-linkers utilized for scaffold preparation include glutaraldehyde, NHS, and EDC, due to their ability to enhance degradation resistance and stabilize scaffolds. Glutaraldehyde, while potent in its stabilization properties, poses challenges in biocompatibility and can induce local cytotoxicity; however, its concentration can be managed [102,103,104]. Examples of scaffolds cross-linked using glutaraldehyde are provided in Table 1, **Entries 2, 7**, and **9**. Meanwhile, the use of EDC improves scaffold efficiency through reducing the availability of cell binding motifs on collagen-like biomaterials [105,106] as demonstrated in Table 1, **Entries 27** and **25**.

Cross-linking is usually verified via spectroscopy (solid-state nuclear magnetic resonance (SS NMR) and Fourier transform infrared spectroscopy (FTIR). The cross-link density of a polymer can be determined with swelling tests through placing the material into water or alcohol at a specific temperature and measuring the change in mass or volume. The higher the extent of cross-linking, the less swelling the material exhibits. Recently, scanning electron microscopy (SEM), particularly secondary electron hyperspectral imaging (SEHI), has been shown to be a useful tool for extracting an SE spectrum for the mapping of semi-crystalline polymers, revealing nanostructure variations and mapping cross-link densities in beam-sensitive biomaterials [107].

## 5. Fabrication Techniques to Generate Porosity

The development of fully functional chitin scaffolds is a multifaceted endeavor, requiring meticulous attention to both microscale and macroscale intricacies. At the microscale level, the scaffold has to establish an environment conducive to the survival and optimal functioning of cells. Meanwhile, at the macroscale, it must orchestrate complex multicellular processes, facilitate the efficient transport of essential nutrients, and possess mechanical properties compatible with the intended application [108]. Central to the fabrication of chitin scaffolds is the judicious selection of appropriate methodologies to generate required material porosity. Porosity has a major effect on facilitating nutrient and oxygen delivery, cell migration governed by the local microenvironment and essential for physiological processes, cell attachment and growth, and the mechanical properties of the scaffold. We refer the reader to the detailed review [109] summarizing the subject of porosity in tissue engineering, focusing on the role of porosity, methods to measure it, and fabrication techniques. The characteristics of chitin scaffolds, ranging from their structural integrity to their degradation kinetics, are intricately intertwined with the fabrication techniques employed.

Despite their promise, conventional scaffold fabrication techniques, which typically entail the construction of porous polymer structures, often deal with achieving the desired complexity in scaffold architecture. Fine-tuning both microscale and macroscale features remains a persistent challenge in conventional methodologies [108,110]. The challenge in chitin-based scaffolds is the insolubility of the polymer; hence, fabrication techniques are limited to 3D printing of a gel [111], sol–gel techniques (including solvent casting and particulate leaching) where porosity is introduced by “salt leaching”, freeze-drying, or gas foaming (supercritical CO_2_ drying).

### 5.1. Rapid Prototyping Method (3D Printing)

The rapid prototyping (RP) method stands out as a scaffold fabrication technique offering a multitude of promising avenues for advancement in tissue engineering. The ability to fabricate three-dimensional features presents opportunities to support the development of extensive tissue formations previously unattainable.

In relation to porous chitinous scaffolds, 3D printing includes extrusion-based printing [112]. The 3D printing of the solution of chitin in [C_2_mim][OAc] IL was investigated by Rogers’ group, and until now that study represents the single published instance of 3D printing of pure chitin [111] without support. A Printrbot Simple Metal 3D printer was used for the printing. The printer was equipped with a heated paste extruder filled with preheated to 40 °C solution of 3 wt% chitin in 1-ethyl-3-methylimidazolium ([C_2_mim][OAc]) IL. The print speed was set to 30 mm/s. As a result, 15 mm height rings with diameters of 20–40 mm and a cube with an edge of 15 mm were successfully printed in a layer-by-layer fashion. The constructs were placed into the water bath, the IL was washed out with repeatable washings, and porosity was induced with freeze-drying techniques. While the printed material was not used as scaffold, the technique is appropriate for scaffold fabrication.

### 5.2. Sol–Gel Technique

Here, a solution of the polymer undergoes a phase transition from liquid to solid at certain critical conditions, called the sol–gel transition. Fundamentally, the sol–gel process orchestrates the conversion of a liquid system, represented by the sol, into a solid state, characterized by the gel. This intricate procedure unfolds in two discernible phases: the solution phase and the gelation phase, where the sol transforms into an interconnected network of solid-phase particles, forming a gel. This is due to the following phenomenon. Polymer solutions of low chitin concentrations are completely isotropic; however, when the polymer concentration increases, the solution transitions to a liquid crystalline gel followed by complete gelation into a solid gel exhibiting an anisotropic structure [79]. Due to the higher extent of the polymer’s chain entanglement, and hence the higher degree of hydrogen bonding, the gel arrangement turns into a more organized structure. Such sol–gel transition of chitin solutions depends on the polymer concentration, temperature, the affinity between polymer and solvent molecules, and the aging time of the chitin solution [113]. The subsequent immersion of gels in various shapes or forms (e.g., bulk, beads, films, fibers) into antisolvents such as water, ethanol, methanol, etc. increases interactions between the polymer chains, reestablishing H-bonding and stabilizing the resulting hydrogel. Such immersion into anti-solvents to reestablish H-bonding is called coagulation. In essence, this is one of the most often employed techniques for the preparation of chitin TE scaffolds.

Many studies have reported the preparation of chitin hydro- or alcogel materials through casting respective solutions in mold, followed by gelation [76,86,87,91]. After washing, hydro- and alcogel were lyophilized or supercritically dried, as discussed in the next section. The type of coagulant and the temperature of the coagulation bath influence the material characteristics of chitin scaffolds. For example, the preparation of chitin gels from CaCl_2_·H_2_O–methanol or CaBr_2_·H_2_O–methanol involves the dissolution of chitin in the solvent (usually for 48 h) to produce chitin gel, keeping it under reduced pressure to remove methanol. The remaining hydrogel is dried under lyophilization [114].

In the realm of tissue engineering, the sol–gel technique has emerged as a cornerstone methodology for scaffold fabrication. Through exploiting its inherent versatility, researchers can tailor scaffolds to meet the diverse requirements of tissue regeneration. Requiring meticulous control over parameters such as composition, porosity, and mechanical properties, sol–gel-derived scaffolds exhibit great promise in facilitating cell growth, proliferation, and tissue integration. Moreover, the ability to incorporate bioactive molecules into the scaffold matrix further enhances its potential for promoting tissue regeneration and functional recovery. As such, the sol–gel technique represents a vital tool in the arsenal of tissue engineers, offering innovative solutions for the restoration and regeneration of damaged or diseased tissues [115,116]. Similar to the previous section, porosity is introduced by a specific type of drying, discussed below.

### 5.3. Solvent Casting and Particulate Leaching

The solvent-casting particulate leaching method [117] represents a significant advancement in scaffold fabrication. This method relies on the casting of a solution of a polymer containing porogens, such as salt particulate. For fabrication, first, the polymer is dissolved in the required solvent (see the **Types of Solvent** section) and then, a porogen is added to the solution, which is transferred to the mold. Here, different salts (mono- and divalent, of different cationic radii) provide the opportunity to tune the microarchitecture (specific surface area, pore size, total pore volume, and pore size distribution) of the resulting hydrogels predictably through varying the salt crystal sizes and their concentration. Once the solution gellifies/solidifies, the porogen is removed via “leaching out” through immersion of the structure into an aqueous bath, which dissolves the salt particles within the matrix. The structures are then dried with the specific drying method discussed below. In Table 1, **Entries 13** and **26** exemplify the use of porogens. Wang et al. utilized sugar in a chitin solution as a porogen. Without the use of a porogen for chitin solutions with concentrations of 0.5. 1.0, and 1.5% (*w*/*w*), the average pore size was <300 µm. However, with the addition of the porogen to a 1.5% chitin solution, the fabricated scaffold exhibited a 1.5 times larger pore size of ~500 µm. Li et al. demonstrated that chitin nanowhiskers–PHVB scaffolds (with PHVB as the main component of the scaffold) using NaCl as a porogen showed a pore size of 9.6 ± 1.8 µm [91].

### 5.4. Gas Foaming Technique and Supercritical Drying

The gas-based technique for scaffold fabrication entails the incorporation of a foaming agent, such as sodium bicarbonate, into the polymer phase. This addition facilitates the generation of an inert gas, typically nitrogen (N_2_) or carbon dioxide (CO_2_). Another technique is supercritical CO_2_ treatment. Biopolymers such as chitin and chitosan in the solid state do not absorb CO_2_. Hence, the CO_2_ foaming of these biopolymers could be conducted in an intermediate hydrogel state (hydrogel foaming). The water in these gels can be replaced with ethanol, and resulting alcogels can be processed with supercritical CO_2_ drying, producing nanoporous materials [118]. Foaming of the hydrogel occurs upon depressurization. This process is also called supercritical drying (SCD) and allows the removal of solvents from a sample while avoiding the collapse of its structure, particularly in porous materials like tissue engineering scaffolds. It operates under conditions where the solvent transitions to a supercritical state, exhibiting properties of both a gas and a liquid.

At a specific combination of temperature and pressure called the critical point, liquid CO_2_ transitions seamlessly between its liquid and gas phases. Above this critical point, the CO_2_ is in a supercritical state, where it has the density of a liquid and the diffusivity of a gas. The sample is typically immersed in a solvent, which infiltrates its pores or interstitial spaces. This step ensures the sample is fully impregnated with the liquid CO_2_. The pressure and temperature of the system are then adjusted to surpass the critical point of CO_2_. This causes the liquid CO_2_ to enter a supercritical state, where it behaves like a gas but maintains a high density. As the supercritical CO_2_ is slowly released from the system, it effectively replaces the liquid solvent within the sample. Importantly, because the CO_2_ is in a supercritical state, there is no surface tension, which typically causes collapse in liquid–gas interfaces. As a result, the sample retains its original structure without shrinkage or distortion. Once the CO_2_ is completely removed, the sample is left in a dry state while maintaining its intricate structure.

The implementation of the supercritical or critical point drying (SCD) technique has significant implications in tissue engineering applications. Utilizing this method, it becomes feasible to achieve optimal preservation of delicate tissue structures. The controlled removal of solvents under supercritical or critical conditions ensures minimal distortion or damage to the tissue architecture.

Limited instances of scaffolds fabricated using supercritical drying techniques are given in Table 1, specifically in **Entries 14**, **18**, and **24**; Tsioptsias et al. utilized supercritical drying techniques to fabricate chitin aerogel, yielding remarkable porosity ranging from 83% to 92%, alongside elevated surface area measurements spanning from 205 to 365 m^2^/g. In their study conducted in 2001, Chow et al. [86] employed supercritical drying techniques to create chitin scaffolds, which exhibited a porosity of 9%. Silva et al. [90] utilized supercritical drying techniques to produce chitin scaffolds, resulting in high porosity ranging from 84% to 90% and a notable surface area ranging from 108 to 145 m^2^/g. Ding et al. [75] utilized supercritical drying techniques to fabricate chitin scaffolds, which demonstrated a surface area ranging from 335 to 366 m^2^/g. Interestingly, compared to the freeze-drying method, the pore size for the supercritically dried scaffolds was in the nanometer range. Specifically, for pure α-chitin, the pore size was in the 2–50 nm range in two instances (**Entries 14** and **24**).

### 5.5. Freeze-Drying

Freeze-drying (also called lyophilization) of a solution, gel, or suspension is a conventional technique to produce porous materials, creating ice crystals that form a microstructure within the scaffold upon ice sublimation. Scaffolds’ topography and arrangement govern cell behavior, proliferation, and differentiation [119]. By subjecting the scaffold to freeze-drying, water within its structure is removed, leading to the formation of interconnected pores. The freeze-drying process preserves the scaffold’s structure while creating a porous network, which is crucial for cell infiltration, nutrient diffusion, and waste removal within the engineered tissue. The degree of porosity can be controlled by adjusting parameters such as freezing rate and drying temperature and duration, allowing the customization of scaffolds with specific porosity levels tailored to the requirements of the target tissue.

Through controlling the temperature of the freezing, it is possible to generate porous materials with different pore sizes, aligned microchannels, and even layered structures. Freeze-drying technology affords precise control over scaffold porosity and morphology, crucial for tissue engineering applications [120]. Optimal scaffolds necessitate uniform interconnected porous structures with specific pore diameters tailored to suit distinct tissue types. Freeze-drying of chitin materials enables fabrication of 3D porous scaffolds with a porosity exceeding 90% and pore diameter that varies up to 500 µm. Specifically, the freeze-drying method involves three steps: 1. preparation of a solution, suspension, or a gel; 2. freezing the prepared solution at different temperatures (−20 °C to −196 °C); and 3. lyophilizing it under high vacuum [121]. For chitin, the material to be freeze-dried exists in the form of either suspension or a gel, due to the polymer’s poor solubility.

Through the manipulation of freeze-drying parameters, scaffold morphology and pore size can be tailored, thereby enhancing biological properties in a targeted manner [122]. The initial stage of freeze-drying is freezing, where the gel (or liquid suspension) of chitin is cooled leading to the formation of ice crystals of pure water. The freezing is conducted in a freezer (−20 °C), using dry ice–organic solvent cooling baths (e.g., dry ice–acetone, −78 °C), or even liquid nitrogen (−196 °C). The faster the material freezes, the smaller the ice crystals that are formed within a sample; smaller crystals mean less damage to the material’s original morphology. Contrastingly, slow freezing produces large ice crystals that “punch through” the material. Freezing conducted in a freezer (−20 °C) is a slow process, whereas freezing using liquid nitrogen (−196 °C) is an ultra-fast process. The important thing to consider when freezing a hydrogel sample is keeping the hydrogel thickness < 2 cm to avoid the danger of pore structure collapse and long drying times.

As freezing progresses, water solidifies. A minimal amount of water remains in a liquid state without freezing (referred to as bound water [123]). During primary drying, which is a crucial step in the process, ice undergoes sublimation. For chitin materials, the sublimation is typically accomplished in a lyophilizer, through lowering the pressure to a level below the water’s triple point [124]. Secondary drying involves the removal of bound water. This phase resembles the final stage of a typical drying process [15,121,125].

The column “*Drying Techniques*” in Table 1 illustrates the conditions for the samples’ freezing. Unfortunately, a few papers did not provide the temperature at which the samples were frozen (Table 1, **Entries 4**, **8**, **10**, **13**, and **21**), which made it difficult to analyze the results. Other entries show that the porosity of the material greatly depends on both the conditions of drying and the composition of the sample. Pure chitin hydrogels frozen at −20 °C (Table 1, **Entry 15**) and then lyophilized exhibited a porosity of 54% and 10 µm pore size. Composite chitin hydrogels with nano-hydroxyapatite as a secondary component (Table 1, **Entries 5**, **6**) were significantly more porous, with a porosity of ~70–80% and large pore size of 250–400 µm; the same porosity was observed for the composite of chitin with silk fibroin (Table 1, **Entry 7**).

Freezing the samples in a dry ice–acetone bath usually provides a temperature of −78 °C. However, if one controls how much dry ice is added, the temperature can be anywhere between −35 and −78 °C. The only example where lyophilization was conducted after freezing at −38 °C was the preparation of composite chitin–nanohydroxyapatite hydrogels; this produced materials with a porosity of 69% and pore size of 200–400 µm (Table 1, **Entry 12**). The comparison between Table 1, **Entry 6** and Table 1, **Entry 12** demonstrates that a lower temperature of freezing resulted in slightly less porous scaffolds while pore size remained the same.

Fast freezing using a dry ice–acetone bath (−38 °C) was used to freeze the following samples before lyophilization: pure chitin (Table 1, **Entry 17**), β-chitin–nanodiopside–nanohydroxyapatite composite (Table 1, **Entry 2**), α-chitin/glass-ceramic composite (Table 1, **Entry 3**), β-chitin/collagen composite (Table 1, **Entry 11**), chitin/sucrose acetate isobutyrate (SAIB) composite (Table 1, **Entry 23**), and β-chitin–gelatin–nanohydroxyapatite (Table 1, **Entry 9**). The presence of other components in the composites makes the comparison challenging, although the comparison of pure chitin hydrogel prepared from DMAc/5% LiCl solvent system, washed, and lyophilized after freezing at −20 °C (Table 1, **Entry 15**) with that frozen at −38 °C (Table 1, **Entry 17**) showed increased porosity of the fast-freeze aerogel (69% vs. 54%). On average, the aerogels obtained after fast-freezing and lyophilization demonstrated a porosity of 62–89%, but the size of pores was about the same as that for aerogels that were slow-dried, with the pore size varying from 126 to 500 µm. The porosity of pure chitin gel that was ultrafast-frozen (−196 °C, liquid nitrogen, Table 1, **Entry 16**) before lyophilization produced material of average porosity, 61%.

Although not often used for chitin scaffolds, it is worth noting the method of directional freezing and ice-templating [126,127]. While conventional freeze-drying produces a scaffold with randomly oriented pores, “directional freezing” allows controlling the direction of freezing, [128,129] producing anisotropic porous scaffolds. Directional freezing has been proposed as a method for the preparation of tissue scaffolds with not only controlled pore dimensions but also directions and can be unidirectional or bidirectional. Unidirectional freezing results in a scaffold with an anisotropic structure in one direction and with several domains of different orientations in another. For this to happen, it is necessary to “direct” water to freeze from only one direction at controlled temperatures, by varying the temperature gradients and hence determining the pore orientation. The elongated pore orientation of the resulting scaffold might be beneficial for certain applications. An additional option is bidirectional freezing [128,129], which requires dual temperature gradients. This causes the ice crystals to spread both horizontally and vertically, resulting in a uniform scaffold structure.

For linking, others have used cross-linkers of different types. The methodologies have also employed various fabrication techniques to introduce porosity into materials. This contributes to the intricate and multifaceted nature of chitin-based scaffolds and affects material porousness, pore size, surface area, and other microarchitectural features of chitin constructs, and, respectively, the performance of materials. In this review, we address these questions individually.

## 6. Cell Types

The important characteristics of scaffolds are their properties such as cytotoxicity, cell attachment, growth, and ability to proliferate. A recent review [130] covers cell lines typically used in regenerative medicine; different cell types serve different regenerative purposes. Importantly, the cells shall be readily available, easy to grow in vitro, and immunocompatible.

Cell studies have been conducted on chitin scaffolds using a large variety of cell lines (Table 2): Epithelial Vero cells (Table 2, **Entries 1**–**3**), preosteoblasts (MC_3_T_3_-E_1_, Table 2, **Entries 4**–**6**), human osteoblasts (MG63, Table 2, **Entries 7**–**11**), human dermal fibroblasts (HDFs, Table 2, **Entries 12**–**15**), human keratinocytes (CRL 2310, Table 2, **Entry 16**), fibroblasts (NIH3T3: Table 2, **Entries 17**–**19**; L929: Table 2, **Entries 20**–**22**; CCL-1: Table 2, **Entry 23**; and CCL-186: Table 2, **Entry 24**); osteoblasts (CRL–427, Table 2, **Entry 25**), human bone cells (CRL-1427, Table 2, **Entry 26**), cultured rabbit chondrocytes (Table 2, **Entry 27**), human adipose stem cells (hASCs and hADSCs, Table 2, **Entries 28** and **29**, respectively), and human osteosarcoma cells (SaOS-2, Table 2, **Entry 30**). There have also been in vivo studies using a rabbit femur model and human mesenchymal stem cells (hMSCs, Table 2, **Entry 31**) with an MTT assay.

In most cases, cell viability assays (the number of live, healthy cells) to detect whether chitin scaffolds display direct cytotoxic effects demonstrated cytocompatibility of either pure chitin or composite scaffolds with living cells. For cell viability experiments, cells are seeded in well plates containing the sterilized scaffolds and the material with no chitin is used as control. The plates are kept for incubation (typically for 24, 48, and 72 h), followed by adding medium with dye and incubation. After that, the optical density of the solution is measured spectrophotometrically using a plate reader. The cell viability graphs indicate the viability of cells attached to the scaffold. All but one study indicated no cytotoxicity or a negligible level. In one instance, indirect cytotoxicity evaluation of α-chitin whiskers–hyaluronan–gelatin scaffolds (Table 2, **Entry 30**), based on the viability of SaOS-2 cells [92], showed that the presence of the chitin nanowhiskers was cytotoxic to cells; however, the following cell attachment and proliferation assays indicated that bone cells were able to attach and proliferate well over scaffold surfaces.

The morphology and spreading pattern of the seeded cells upon attachment to the scaffolds are typically assessed with staining (e.g., 4,6-diamidino-2-phenylindole (DAPI)). In most of the studies, cell attachment and proliferation assays have confirmed well-improved cell attachment (compared to no-chitin control), spreading, and formation of a monolayer of cells on the surfaces of the scaffolds, confirming enhanced cell proliferation.

There has also been an in vivo study [32] of mesenchymal stem cell (MSC)-loaded HA–chitin matrixes, in which green fluorescence protein (GFP) transfected MSC-induced osteoblasts were loaded onto porous HA–chitin matrixes and intramuscularly implanted into a rabbit femur. The cell-loaded HA–chitin scaffold (and nonporous film as a control) was press-fitted into the rabbit bone defect. It was found that the HA–chitin scaffold did not induce any acute inflammatory response after 14 days. The observations 2 months after implantation suggested that both cell-free and cell-loaded porous HA–chitin matrixes promoted the ingrowth of surrounding tissues, with the cell-loaded HA–chitin matrixes being the better performers.

## 7. Degradation

The biodegradability of the scaffold is an important factor to consider during the design of the scaffold, as the scaffold represents only temporary support.

Upon the contact of chitinous hydrogels with water, the chitin chains swell, resulting in spatial changes in the chain positions and loosening of H-bonding. It can be expected that, due to less significant H-bonding, β-chitin is more susceptible to biodegradation than α-chitin with a similar %DA value [131]. Chitin degradation involves three steps: the initial hydrolysis of the (1→4)-β-glycoside bond (hydrolysis), deacetylation, and deamination [132]. Here, chitin can be hydrolyzed into smaller Mw species, deacetylated to chitosan, and then subjected to deamination, forming cellulose-like forms.

Regarding %DA values, the question is somewhat controversial in the literature. Some studies of the influence of the %DA on enzymatic degradation showed that higher %DA possessed a lower affinity for the enzymes and, hence, higher %DA chitins exhibited a slower degradation rate [131]. Other studies reported that the opposite was true and showed that the degradation rate increased with increasing %DA [133]. Thus, study [134] showed that the initial degradation rate was only about 0.2%/day for chitin with %DA 7, 19, and 29, respectively, whereas the degradation rate increased to 0.8%/day, 3.0%/day, and 7.8%/day, respectively, for chitin with %DA 38, 44, and 48%. That study agrees with a work performed by Tomihata and Ikada [135] who studied the degradation of chitin films and found that the in vivo biodegradation rate experienced a dramatic increase when the %DA increased to 27%.

Overall, it appears that the degradation rate increases to a certain value with a decrease in %DA and shows a maximum at %DA of about 50%. The rate then decreases, and the fully deacetylated chitosan with %DA = 3% is degraded very slowly [131]. In respect to Mw, the results of degradation studies showed that a higher molecular weight of chitin decreases its degradation rate [134].

Biodegradability has to be balanced with adequate mechanical properties of the scaffolds, and it is a catch-22 between fast degradation and strong mechanical properties. Furthermore, the controlled degradation allows slow delivery of drugs or growth factors (if loaded into the matrix). The degradability of the scaffolds is quantified according to the following equation:

Degradability (%) = W_t_/W_i_ × 100%, (1)

where W_i_ is the initial dry weight of the scaffold and W_t_ is the dry weight of the specimens after each respective in vitro degradation assay.

However, not in all cases biodegradability has been assessed. Thus, the degradability of *α*-chitin whiskers/hyaluronan/gelatin scaffold was assessed under three conditions [92]: 1. individual scaffold immersion into a 10 mM phosphate buffer saline (PBS) solution (pH 7.4) at room temperature without shaking for 24 h; 2. individual scaffold immersion into a 10 mM PBS solution at body temperature (37 °C) under shaking (70 rpm) for 24 h; and 3. individual scaffold immersion into a bacterial collagenase (COL) solution at 37 °C under shaking. After the specified time, the specimens were removed from the media, frozen at −40 °C, and lyophilized. It appears that a large amount of chitin in the scaffold enhanced the resistance to degradation. At RT, with no shaking, the remaining weights of the scaffolds were about 58–76% of their original dry weights. At 37 °C, 45–50% of the scaffolds degraded in PBS solution, whereas in the COL medium, the degradation of the scaffolds was significantly higher (~60%). Regardless of the CW content, the values after 24 h were in the range of 11–52% of the original dry weights of the scaffolds.

An in vitro *α*-chitin–pectin–CaCO_3_ scaffold degradation study [60] showed that the scaffold degraded slowly till 14 days and much faster thereafter, demonstrating a degradation of ~60% at day 21. In the presence of lysozyme, the pectin–chitin matrix degraded gradually in a controlled manner, and the degraded products were found to be beneficial to the human body.

In vivo studies of an *α*-Chitin–nanohydroxyapatite scaffold [32] demonstrated that after 90 days, the implants were completely loosened compared with the control, and the scaffold no longer formed a cohesive matrix releasing hydroxyapatite particles. It was noted that the degradation of the chitin–HA materials in the preliminary study was somewhat too rapid.

## 8. Conclusions

Material selection for scaffolds for tissue engineering is now moving towards natural components rather than synthetic polymeric materials. Due to chitin’s structure being similar to that of GAG, constituting the major component of ECM, the polymer can regulate cell behavior during tissue regeneration and promote cell attachment, proliferation, and differentiation. Furthermore, its therapeutic properties, including analgesic, antitumor, non-toxic, non-allergenic, hypocholesterolemic, and hemostatic activity, underscore its potential in biomedical applications. The most crucial property of chitin is its biodegradability, as it typically undergoes degradation in the human body within ~12 weeks post-surgery. However, during chitin processing, there is a need for precise control to achieve the desired microarchitectural features such as porousness, pore size, surface area, etc. In essence, tissue engineering (TE) scaffold fabrication from chitin involves careful consideration of different factors including chitin types, solvents, cross-linkers, and fabrication techniques.

The selection of chitin types, whether *α*-chitin or *β*-chitin, and their forms, either microcrystalline or nanocrystalline, can have a significant impact on the physicochemical and biological properties of the resulting scaffolds. Additionally, parameters of chitin like degree of acetylation (%DA) and deacetylation pattern have a significant influence on porousness, pore size, and surface area. The molecular weight of the polymer defines the material’s mechanical strength and extent of chain entanglement, which is important for TE scaffold porosity.

Overcoming the insolubility of chitin due to extensive polymer hydrogen bonding presents a challenge. Various types of solvents such as inorganic bases, calcium salts, and lithium salts can dissolve chitin. However, they do not apply to TE due to the challenges associated with their removal. Perfluorinated systems are also not suitable due to their toxicity. Solvent systems like CaCl_2_–methanolic solution, (DMAc)–LiCl, sodium hydroxide–urea aqueous eutectic (8 wt% NaOH/4 wt% urea/88% water), ionic liquids (ILs), and deep eutectic solvents are currently solvents of choice in TE scaffolding.

Achieving desired mechanical properties and degradation resistance necessitates precise control over cross-linking methods, whether physical or chemical (e.g., glutaraldehyde, NHS, EDC). Since the scaffold offers only temporary support, the cross-linkers should be non-toxic, and there needs to be a careful balance between the improvement of mechanical properties and the rate of degradation.

Fabrication techniques such as sol–gel techniques (including porogen leaching), freeze-drying, and supercritical drying (Sc-CO_2_) are important in making scaffolds with desired morphological properties and porosities. To illustrate, the solvent-casting particulate leaching method involves the casting of a polymer solution with a porogen (e.g., salt, sugar). On the other hand, supercritical drying involves the removal of solvents and the transition of the solvent to a supercritical state. Meanwhile, the freeze-drying technique forms a porous network by keeping the original shape of the structure. Porosity is adjusted through changing the temperature of freezing and drying conditions.

Our review emphasizes the potentiality of chitin and the pressing need for further research to overcome existing challenges and fully harness its capabilities in tissue engineering. Through addressing the intricacies of chitin-based scaffold development, we can advance toward more effective strategies for tissue regeneration and biomedical applications.

## Figures and Tables

**Table 2:** Cell studies conducted on chitin-containing scaffolds (grouped by cell type).

Entry	Scaffold	Cells	Result	Ref
1	*β*-Chitin (%DA = 72.4)–nanosilver *β*-Chitin–nanohydroxyapatite	Vero cells (epithelial): A mammalian cell line	Cell attachment studies using vero (epithelial) cells showed that the cells were well attached to the scaffolds.	[81]
2	*α*-Chitin–nanohydroxyapatite		Cells were found to attach and spread on the scaffolds.	[57]
3	*β*-Chitin–nanohydroxyapatite		The cell viability, attachment, and proliferation studies confirmed the cytocompatibility of scaffolds with well-improved cell attachment and proliferation.	[82]
4	*β*-Chitin–nanodiopside–nanohydroxyapatite	MC_3_T_3_-E_1_: A mouse preosteoblast cell line	Cell studies proved the cytocompatibility of the composite scaffolds with improved cell adhesion.	[58]
5	*β*-Chitin–silk fibroin–mesoporous silicate		Cell studies proved the cytocompatible nature of the composite scaffolds with well-improved proliferation and cell attachment.	[61]
6	*β*-Chitin–gelatin–nanohydroxyapatite		Cell studies demonstrated the cytocompatibility nature of the composite scaffolds.	[62]
7	*α*-Chitin–nanobioactive glass ceramic	MG63: A human osteoblastic cell line (osteoblastic model)	Cell attachment studies indicated no sign of toxicity, and cell attachment to the pore walls.	[59]
8	*α*-Chitin–nanosilica		Biocompatible when tested with MG 63 cell line.	[31]
9	*α*-Chitinnanohydroxyapatite		Cells were found to attach and spread on the scaffolds.	[57]
10	*β*-Chitin–nanohydroxyapatite		The cell viability, attachment, and proliferation studies confirmed the cytocompatibility of scaffolds with well-improved cell attachment and proliferation.	[82]
11	*α*-Chitin–pectin–CaCO_3_ nanopowder		Negligible toxicity towards cells. Cell attachment and proliferation studies showed that cells attached to the scaffolds and started to proliferate after 48 h of incubation.	[60]
12	*α*-Chitin–nanohydroxyapatite	Human dermal fibroblasts (HDFs)	Cells were found to attach and spread on the scaffolds.	[57]
13	*β*-Chitin–nanohydroxyapatite		The cell viability, attachment, and proliferation studies confirmed the cytocompatibility of scaffolds with well-improved cell attachment and proliferation.	[82]
14	Chitin–PHBV		Showed enhanced HDF cell attachment and proliferation.	[63]
15	*β*-Chitin–collagen		Fibroblasts were attached to collagen-coated scaffolds, whereas cells did not attach and aggregate on the scaffold of chitin alone.	[83]
16	Mycelial mats: *α*-Chitin–β-glucan	CRL 2310: Human keratinocyte cell line	Scaffolds seeded with keratinocytes showed deposition of extracellular matrix (ECM) components and the formation of cell sheets in 14 days.	[88]
17	*α*-Chitin–nanohydroxyapatite	NIH3T3: A fibroblast cell line	Cells were found to attach and spread on the scaffolds.	[57]
18	*β*-Chitin–nanohydroxyapatite		The cell viability, attachment, and proliferation studies confirmed the cytocompatibility of scaffolds with well-improved cell attachment and proliferation.	[82]
19	*α*-Chitin–pectin–CaCO_3_ nanopowder		Negligible toxicity towards cells. Cell attachment and proliferation studies showed that cells attached to the scaffolds and started to proliferate after 48 h of incubation.	[60]
20	*α*-Chitin	L929: A fibroblast cell line	Fibroblast cells were well attached to the chitin gels and maintained their normal morphologies compared with controls in normal culture plates.	[75]
21	*α*-Chitin		Produced materials had deficient cytotoxicity levels.	[90]
22	*α*-Chitin–pectin–CaCO_3_ nanopowder		Negligible toxicity towards cells. Cell attachment and proliferation studies showed that cells attached to the scaffolds and started to proliferate after 48 h of incubation.	[60]
23	*α*-Chitin–nanohydroxyapatite	CCL-1: Mouse fibroblasts	HA–chitin materials were non-cytotoxic.	[32]
24	*α*-Chitin–nanohydroxyapatite	CCL-186: Human lung fibroblast	HA–chitin materials were non-cytotoxic.	[32]
25	*α*-Chitin–nanohydroxyapatite	CRL-427: Human osteoblasts	Cells adhered, spread, and formed a monolayer on the surfaces of the matrixes, confirming cell proliferation.	[32]
26	*α*-Chitin–nanohydroxyapatite	CRL-1427: Human bone cell	HA–chitin materials were non-cytotoxic.	[32]
27	*β*-chitin sponge with a cartilage-like layer at its surface	Cultured rabbit chondrocytes	Culturing of cells directly with scaffold did not promote any visible cell damage. The cell layer at the surface of the *β*-chitin sponge was filled with chondrocytes and abundant extracellular matrix.	[87]
28	*α*-Chitin (%DA = 57.9)–sucrose acetate isobutyrate (SAIB)	hASC (also hADSC): Human adipose stem cells	The cells were able to spread in the scaffolds. Scaffolds were able to support cell viability and proliferation in culture with an osteoblastic cell line. Cell proliferation rates increased after 24 h, decreasing after 48 h. After 72 h of culture, cell proliferation improved. After 72 h of culture, cells were wholly adapted.	[89]
29	Chitin nanocrystals–poly(3-hydroxybutyrate-co-3-hydroxy valerate) (PHBV)		Scaffolds enhanced hADSC adhesion.	[91]
30	*α*-Chitin whiskers–hyaluronan–gelatin	SaOS-2: Human osteosarcoma cells	The presence of the CWs (at 30%) was cytotoxic to cells; however, observation indicated that bone cells attached and proliferated well over scaffold surfaces.	[92]
31	*α*-Chitin–nanohydroxyapatite	MSCs: Mesenchymal stem cells in vivo rabbit femur model	Both cell-free and cell-loaded porous HA–chitin matrixes promoted the ingrowth of surrounding tissues, with the cell-loaded HA–chitin matrix being the better performer.	[32]

## Data Availability

Data sharing is not applicable.
